# Life-Threatening Anaphylaxis due to Cerebrolysin®

**DOI:** 10.1155/2024/2332908

**Published:** 2024-07-18

**Authors:** Helmut Trimmel, Wolfgang Tauber, Martin Zikeli

**Affiliations:** ^1^ Department for Anesthesia Emergency and Intensive Medicine State Hospital of Wiener Neustadt, Corvinusring 3-5, Wiener Neustadt 2700, Austria; ^2^ Karl Landsteiner Institute for Emergency Medicine Medical Simulation and Patient Safety State Hospital of Wiener Neustadt, Corvinusring 3-5, Wiener Neustadt 2700, Austria; ^3^ Danube Private University Department of Medicine, Steiner Landstraße 124, Krems an der Donau 3500, Austria; ^4^ Central Laboratory State Hospital of Wiener Neustadt, Corvinusring 3-5, Wiener Neustadt 2700, Austria; ^5^ Department for Dermatology and Venereology State Hospital of Wiener Neustadt, Corvinusring 3-5, Wiener Neustadt 2700, Austria

## Abstract

In this case report, we describe a well-documented, severe anaphylactic reaction after intravenous administration of cerebrolysin, a neurotrophic agent derived from highly purified porcine brain tissue, consisting of peptides and free amino acids. Cerebrolysin has been in use for decades, in various neurological diseases, but especially stroke and traumatic brain injury, with the aim of enhancing cognitive performance. After administration of cerebrolysin to an 85-year-old male patient suffering from subacute stroke, he developed a fulminant anaphylactic reaction. Following institutional standards, vital functions were quickly restored. The anaphylactic reaction was clearly confirmed by laboratory tests. To date, only rare cases of anaphylaxis due to cerebrolysin have been published in the literature. The current report is intended to raise awareness for the possibility of such a reaction, given the widespread use of cerebrolysin in several indications in mostly critical patients. The case shows how a completely unexpected life-threatening situation can be successfully treated by targeted measures, if the situation is recognized quickly. In light of this event, we consider pathophysiology of allergic reactions and treatment guidelines.

## 1. Introduction

Insect stings and drugs are the primary anaphylaxis-causing substances in adults [1]. Intravenous administration in particular can lead to severe reactions in predisposed individuals. Severe anaphylactic reactions are life-threatening; respiratory failure and circulatory collapse can lead to death within minutes. Therefore, knowledge of the allergenic potential of medications is essential in order to quickly recognize physical reactions. The allergenic potential of antibiotics or analgesics, for example, is well known, while other medications are classified as safe based on approval studies and often decades of experience. There are only a few precise epidemiological studies on the frequency of drug hypersensitivity [2]. Here, we report on a drug for which there is hardly any allergy experience to date. Our findings indicate that a severe type I reaction occurred.

## 2. The Case

The case involves an 85-year-old male patient with multiple pre-existing morbidities, including type II diabetes, hypertension, coronary heart disease, severe aortic stenosis, and a bipolar disorder with decades of use of psychotropic drugs. In addition, he had suffered from recurrent episodes of erysipelas for more than 5 years. The patient reported intolerance reactions to acetylsalicylic acid and a cephalosporin with mild skin reactions that had occurred several years ago. The reason for the current inpatient admission was an episode of spondylodiscitis, possibly related to the skin infection. On day 18 after admission, the patient suffered an ischemic stroke, though blood pressure and oxygenation remained stable. At this time, a comprehensive drug therapy was established (Table 1) [3]. CT angiography (CTA) performed immediately verified vascular occlusion in the M2 segment of the left art. CT angiography (CTA) performed immediately verified vascular occlusion in the M2 segment of the left art. cerebri media at the insula cortex level with an extensive prefusion deficit of the middle and rear media. The patient received intravenous thrombolysis (alteplase, 90 mg) followed by thrombectomy under general anaesthesia. The reperfusion result was classified as Tici 2b [3]. The event may be classified as embolic stroke of undetermined source (ESUS), since atrial fibrillation was never detected and transesophageal echocardiography did not reveal a relevant pathology except for moderate- to high-grade stenosis of the aortic valve but without thrombotic deposits [4, 5]. Unfortunately, no further diagnostics, e.g., of the carotid arteries were performed. Clinically, motor aphasia with partially preserved language comprehension, a severe right-side hemiparesis (most pronounced on the arm), and hemihypesthesia persisted; the patient also had swallowing disorders which led to pulmonary aspiration. Because of the latter, antibiotic therapy was switched to piperacillin/tazobactam (3 × 4.5 g) and fosfomycin (3 × 8 g) on day 20 (day 2 after stroke).

Due to increasing respiratory insufficiency, the patient was transferred from the stroke unit to the intensive care unit (ICU) on day 23, five days after the stroke. The cerebral and thoracic CT scan performed just before ICU admission revealed a slight hyperdense imbibition in the area of the gyrus pre- and postcentralis, as well as a (newly arisen) lack of frontal cortex differentiation and slightly enlarged internal and external CSF spaces. The thorax scan revealed signs of pneumonia with pleural effusions and concomitant atelectasis on both sides. Radiopaque material in the trachea obliterated the left main bronchus. Vasosclerosis, including coronary sclerosis, was also described.

On ICU admission, a bronchoscopy was done immediately to clear the obstructing secretion from the bronchus, and a right-side pleural drainage removed 1,000 ml of amber effusion.

Now, with significantly improved hemodynamic and respiratory status, the patient received 30 ml of cerebrolysin as a bolus infusion, according to the institutional stroke therapy standard, at 6:13 pm. Approximately 30 minutes later, he suffered a hemodynamic collapse, initially as supraventricular tachycardia (*f* = 103/min). Blood pressure fell from 125/78 (MAP 94) to 58/26 (MAP 38), and oxygen saturation dropped to 89% due to severe bronchospasm. Oxygen, fluids, and a vasopressor were administered as immediate symptomatic measures; shortly after that, an anaphylactic reaction was assumed as probable and therapy was adapted accordingly (details below). Nevertheless, the patient developed ventricular tachycardia with a heart rate of up to 175/min, which could only be controlled by 300 mg amiodarone. The severity of the attack was graded as III°.

During the ongoing treatment, the team also reviewed possible triggers. Ultimately, the link to the time of the cerebrolysin administration was indicative because there had been no other changes in drug application during the previous 24 hours. Though it was not possible to interrupt the allergen intake, since the entire prescribed dose had already been administered, the therapy succeeded in stopping the reaction. The hemodynamic depression and the respiratory impairment were resolved in less than 30 minutes. Within 3 hours, all clinical symptoms except the exanthema had subsided; approximately ten hours after the event, the patient no longer needed vasoactive drugs. No further allergic symptoms occurred during the further clinical course. A single episode of ventricular tachycardia was sustainably stopped with 150 mg amiodarone the day after the anaphylactic event. After this, there were no longer any cardiac arrhythmias that required treatment.

### 2.1. Diagnostic Assurance

**Figure 1:**
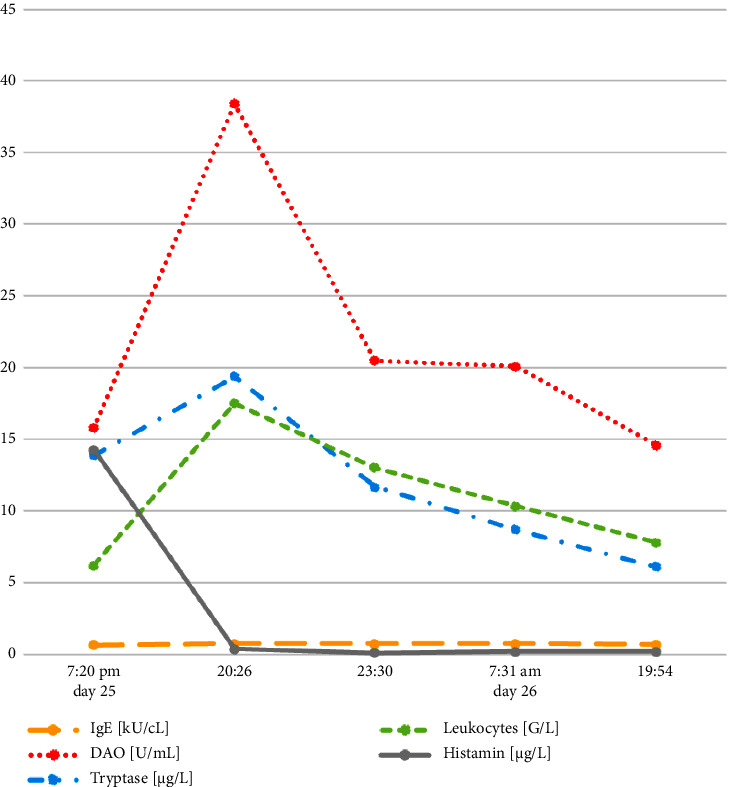
Laboratory findings. Graphical representation of the progression of the relevant laboratory parameters. For more detailed information, see Supplementary Table 1.

**Figure 2:**
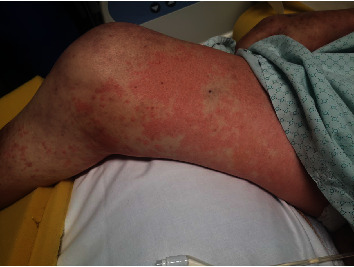
Exanthema on the right lower limb. Depiction of the patient's maculopapular rash in the area of the right upper and lower leg.

To confirm the clinical diagnosis, the institutionally specified “anaphylaxis lab block” was done. This includes differential blood count, IgE, tryptase, and interleukin-6, as well as histamine and di-amino oxidase (DAO). Blood samples were taken one, two, five, 13, and 25 hours after the event. The laboratory results confirmed the anaphylactic reaction, with a sudden increase of the tryptase value immediately after the event and a rapid decrease hours later (Figure 1). Histamine and DAO showed a similar course, and the leukocytes also reflected the stress reaction. Also, striking was an urticarial rash occurring a few minutes after the acute event, which showed not only on the trunk but also on the extremities (Figure 2). Though it was not possible to carry out an allergological test such as a radio-allergo-sorbent test (RAST) or skin testing, the significant increase in tryptase, ideally measured 1–3 hours after the event, as well as the histamine level, indicates a mast cell-mediated reaction. There is a correlation between the severity of anaphylaxis and the extent of the increase in tryptase.

### 2.2. Therapy of Anaphylaxis

In the acute phase, the patient received oxygen with high flow (15 l/min) via a face mask, 1,500 ml of balanced whole electrolyte solution (Elomel isoton®), noradrenaline in increasing dosage via perfusor (max 0.8 mcg/kg/min), 60 mg Dibondrin® (diphenhydramine-hydrochloride), and 40 mg of Fortecortin® (dexamethasone). The fluids balance of this day was finally +646 ml.

Noradrenaline was chosen as a vasopressor by the ICU physician because the patient had pronounced tachycardia and it was not clear initially whether it was caused by atrial fibrillation or sinus arrhythmia. The predominantly *α*-mimetic agent noradrenaline is less positively chronotropic and therefore less disruptive to coronary perfusion but counteracts vasoplegia as well as adrenaline. However, adrenaline functionally inhibits all the important pathomechanisms of anaphylaxis via the activation of *α*- and *β*-adrenoceptors: vasoconstriction, reduction of vascular permeability and edema formation, and bronchodilation, and it also increases inotropy [6–8]. Therefore, it should always be the vasopressor of first choice, since anaphylaxis-related tachycardia tends to decrease in frequency after adrenaline administration. In the case described here, immediate intravenous application of 80 *µ*g (=1 *µ*g/kg BW) would have been the most suitable dose. Intramuscular administration was not considered because a central venous catheter was already in place, guaranteeing rapid and predictable delivery.

Anaphylaxis is usually caused by an immunological reaction, most often immunoglobulin-E-mediated. IgE activates mast cells and basophil granulocytes that release histamine, prostaglandins, leukotrienes, tryptase, proteases, serotonin, and cytokines. As histamine may play a central role in anaphylaxis [9], H_1_ antagonists are recommended; H_2_-blockers cannot be used because they are not available in a parenteral formulation in Austria. Glucocorticoids, despite having a slow onset of action in the acute phase and lacking systematic clinical studies in anaphylaxis, can be considered based on their membrane-stabilizing effect [10].

### 2.3. The Trigger

The triggering agent in this episode, cerebrolysin, is a neuroprotective and neurotrophic drug that has been used for many years in the treatment of various neurological diseases, especially for patients with cerebral insults. The drug is obtained from highly purified (porcine) brain proteins by standardized enzymatic degradation and consists of 25% low molecular weight peptides and free amino acids. Using immunoassay (ELISA), several fragments of neurotrophic factors were identified that stimulate neurotrophic signaling pathways and thus promote the growth, differentiation, and survival of nerve cells [11–13]. Although still controversial [14], improvements in cognitive performance were shown in prospective, randomized studies [15–17]. Cerebrolysin is provided by EVER Pharma Ltd., 4866 Unterach, Austria, and is currently approved in Austria, China, Germany, Russia, and South Korea.

Possible triggers in this case would include an alpha-gal reaction or serum albumin allergy based on the mammalian origin of the drug. Cat epithelia-serum albumin cross-reaction is also known. However, none of these types of reaction have been reported for cerebrolysin. In the context of anaesthesia or intensive medical treatment, anaphylactic reactions are occasionally caused by hidden allergens, such as chlorhexidine in the antiseptic coating of venous cannulas or components of lubricant for placing urinary catheters, and polyethylene glycol as an excipient in numerous drugs.

## 3. Discussion

In this case, a severe adverse reaction to cerebrolysin occurred, which fortunately responded well to immediate, guideline-oriented therapy [6–8, 18]. Over decades, cerebrolysin has accumulated a good safety record, as evidenced by clinical use, observations from postmarketing surveillance studies, and safety data from randomized, controlled clinical trials. The reported events showed that side effects are generally mild and temporary. The most common adverse events were dizziness, headache, skin flushing, nausea, and systemic symptoms such as fatigue and insomnia. A recent update of the Cochrane review on cerebrolysin in acute ichaemic stroke-indicated moderate certainty of evidence for an increase in nonfatal serious adverse events with cerebrolysin use, in particular for a dose of 30 ml/day [19]. Interestingly, the study by Strilciuc et al. indicated that compared to the placebo, the highest dose of cerebrolysin (50 mL, as we use it in sTBI) had the lowest occurrence rate of SAE and nonfatal SAE, with a risk reduction of >25%, thus suggesting some effects of the agent against adverse events [16]. Nevertheless, in controlled clinical trials as well as recent meta-analysis, the frequency of adverse events was quite similar in the groups treated with cerebrolysin and placebo [20, 21]. It may be noteworthy that anaphylaxis was not explicitly reported in any of the RCTs cited in the meta-analysis. To our knowledge, there are only two publications so far on life-threatening allergic reactions. In a literature review from China, Zhao-bao lists 35 cases from 1994 to 2009 with anaphylactic reactions within minutes to hours after administration of cerebrolysin [22]. In 23 cases, this occurred after the first and in 12, after the second dose. One patient died as a result of shock. The paper recommends greater caution when delivering the drug. A second paper, also from China, reports on a single patient with severe allergic shock, including the need for cardiopulmonary resuscitation [23]. The patient was, however, discharged from hospital without any neurological damage.

When specifically asked by the authors, the manufacturer said they had no knowledge of any exact data on severe allergic reactions. The package leaflet of cerebrolysin points to the possibility of allergic reactions: special caution is recommended in allergic diathesis, and very rare (<1/10,000) allergic or hypersensitivity reactions are mentioned, such as tingling, skin and local vascular reactions, neck, head and limb pain, fever, mild back pain, shortness of breath, chills, and “shock-like condition.”

Cerebrolysin can still be considered a safe drug, but in the context of the first and also second application, and especially in patients with allergic diathesis, increased caution may be required. At least it should be administered in settings where knowledge of severe allergic reactions and a standardized treatment plan are available. It should be noted that in most cases, cerebrolysin is not administered in intensive care units, where medical knowledge, the presence of well-trained doctors, and the ability to act are likely to be greater than in a normal ward. And yet, when it comes to severe allergic reactions, every minute may count.

## Figures and Tables

**Table 1:** Patient's long-term medication provided at the time of the stroke.

Cefazolin 2g 1-1-1 (since hospital admission)
Candesartan HCT 16/12,5 mg 1-0-0
Atorvastatin 10 mg 0-0-1
Melperon-HCl 25 mg 0-0-2-1
Procyclidin 5 mg 1-0-0
Escitalopram 10 mg 1-0-0
Cariprazinhydrochlorid 1.5 mg 1-0-0
Quetiapin 25 mg 0-0-1
Pantoprazol 40 mg 1-0-0
Transdermal fentanyl patch 25 *µ*g/h
Hydromorphonhydrochlorid 2.6 mg (rescue)
Metamizol 500 mg 1-1-1-1
Diclofenac 50 mg 1-0-1
Folsan 5 mg 1-0-0
Colecalciferol 20.000 IU 1/week (Mo)
Prostaurgenin 0-0-1
Movicol (polyethylene glycol 3350 with electrolytes) 1-1-0 bags
Enoxaparin sodium 40 mg 0-0-1 sc

g: gram; HCT: hydrochlorothiazide; mg: milligram; *µ*g: microgram; IUs: international units; sc: subcutaneously.

## Data Availability

All data generated or analyzed during this study are included within this article. Further enquiries can be directed to the corresponding author.

## Supplementary Materials

Supplementary Materials Supplementary Table 1: complete list of laboratory values over time.
