# Supplementary material for: Life-Threatening Anaphylaxis due to Cerebrolysin®
Source: Case Rep Neurol Med. 2024 Jul 18;2024:2332908. doi: 10.1155/2024/2332908 (PMC11272398; doi:10.1155/2024/2332908)
Supplement: Supplementary Materials — Supplementary Table 1: complete list of laboratory values over time. [file 2332908.f1.docx]

Supplement table 1: List of laboratory values over time

| Date | 28.10.23 | | | | 29.10. | |  |  |
| --- | --- | --- | --- | --- | --- | --- | --- | --- |
| Time | 12:47 | 19:20 | 20:26 | 23:30 | 07:31 | 19:54 | *Range* | *Unity* |
| Leukocytes | 8.17 | 6.20 | 17.53 | 13.01 | 10.35 | 7.79 | *3,6-10,5* | *G/L* |
| Eosinophilic granulocytes abs. | 0,03 | 0.06 | 0.23 | 0.02 | 0 | 0.26 | *0.02-0.5* | *G/L* |
| Eosinophilic granulocytes rel. | 0.40 | 1.0 | 1.3 | 0.2 | 0 | 3.3 | *0.5-5.5* | *%* |
| Basophile granulocytes abs. | 0.09 | 0.02 | 0.08 | 0.05 | 0.03 | 0.07 | *0.0-0.2* | *G/L* |
| Basophile granulocytes rel. | 1.10 | 0.30 | 0.5 | 0.4 | 0.3 | 0.9 | *0.0-1.75* | *%* |
| Monocytes abs. | 0.55 | 0.40 | 0.71 | 0.42 | 0.45 | 0.6 | *0.1-0.9* | *G/L* |
| Monocytes rel. | 6.70 | 6.50 | 4.1 | 3.2 | 4.3 | 0.9 | *2-9.5* | *%* |
| IgE |  | 67.9 | 76.2 | 74.9 | 77.7 | 70.4 | *10-100* | *U/ml* |
| Histamine |  | 14.3 | 0.4 | <0.1 | 0.2 | 0.2 | *< 1.0* | *mcg/L* |
| DAO |  | 15.8 | 38.4 | 20.5 | 20.1 | 14.6 | *> 10.0* | *U/ml* |
| Tryptase |  | 13.9 | 19.4 | 11.7 | 8.7 | 6.1 | *11.4* | *mcg/L* |
